# Supplementary material for: Seasonal changes in fish assemblage structure at a shallow seamount in the Gulf of California
Source: PeerJ. 2016 Sep 6;4:e2357. doi: 10.7717/peerj.2357 (PMC5018667; doi:10.7717/peerj.2357)
Supplement: Supplemental Information 1 — Log likelihood and Chi-square statistical probabilities for multiple logistic regression of abundance, using ordinal values on sea surface temperature (SST) and subsurface temperature (T30) for 17 species occurring at EBES. [file peerj-04-2357-s001.pdf]

Supplemental Table S1. Log likelihood and Chi-square statistical probabilities for multiple logistic regression of abundance, using ordinal values on sea surface temperature (SST) and subsurface temperature (T30) for 17 species occurring at EBES.

				SST				T30			
Species	- Log likelihood	ChiSquare	Prob>ChiSq	Estimate	Std Err	ChiSquare	Prob>ChiSq	Estimate	Std Err	ChiSquare	Prob>ChiSq
A. solandri	1.33	2.654	0.2653	-0.028	0.190	0.02	0.8832	-0.252	0.229	1.22	0.2695
C. caballus	10.31	20.629	0.0001	-0.765	0.228	11.28	0.001	0.245	0.199	1.53	0.2165
C. hippurus	3.38	6.751	0.0342	-0.387	0.279	1.92	0.1660	-0.154	0.246	0.39	0.5318
D. macarellus	1.11	2.215	0.3305	-0.246	0.169	2.12	0.1454	0.217	0.214	1.02	0.3127
E. lineatus	1.53	3.054	0.2172	-0.226	0.147	2.37	0.1235	0.292	0.187	2.44	0.1181
L. argentriventris	3.04	6.086	0.0477	-0.147	0.145	1.03	0.3091	-0.191	0.189	1.03	0.3111
L. colorado	1.44	2.889	0.2359	-0.266	0.177	2.26	0.1329	0.128	0.199	0.41	0.5205
L. guntheri	2.08	4.154	0.1253	0.034	0.138	0.06	0.8035	-0.297	0.182	2.67	0.1022
L. novemfasciatus	3.78	7.552	0.0229	-0.016	0.180	0.01	0.9290	-0.440	0.229	3.69	0.0549
L. peru	4.16	8.312	0.0157	-0.152	0.150	1.03	0.3112	0.552	0.223	6.12	0.0134
M. jordani	4.07	8.138	0.0171	0.120	0.152	0.62	0.4302	0.357	0.218	2.67	0.1020
M. rosacea	0.68	1.365	0.5052	0.055	0.139	0.16	0.6916	0.102	0.175	0.34	0.5594
P. colonus	0.40	0.804	0.6690	0.214	0.273	0.61	0.4329	-0.081	0.297	0.07	0.7848
S. lalandi	8.72	17.443	0.0002	0.611	0.233	6.87	0.0088	0.322	0.316	1.04	0.3078
S. rivoliana	8.42	16.837	0.0002	0.013	0.149	0.01	0.9332	0.698	0.248	7.93	0.0049
S. lewini	5.74	11.482	0.0032	0.409	0.169	5.84	0.0156	-0.700	0.233	9.01	0.0027
T. albacares	0.24	0.482	0.7860	-0.120	0.197	0.37	0.5426	0.154	0.245	0.39	0.5298
